# Associations between diet quality, food insecurity, physical activity, social connectedness and depressive symptomology in Australian university students: a cross-sectional study

**DOI:** 10.3389/fnut.2025.1692643

**Published:** 2025-11-05

**Authors:** Simone O’Neill, Michelle Minehan, Catherine R. Knight-Agarwal, Vivienne Lewis, David B. Pyne

**Affiliations:** ^1^Research Institute for Sport and Exercise, Faculty of Health, University of Canberra, Canberra, ACT, Australia; ^2^Faculty of Health, University of Canberra, Canberra, ACT, Australia

**Keywords:** mental health, major depressive disorder, diet quality, community networks, food insecurity

## Abstract

**Objective:**

Rising prevalence of depression demands focus on modifiable factors to improve mental health outcomes. An inverse relationship has been identified between diet quality and depressive symptomology. This cross-sectional study aimed to examine how food insecurity, physical activity and social connectedness contribute to the relationship between diet quality and depressive symptomology.

**Methods:**

Data was collected from adult university students (*n* = 145, age = 26.9 ± 9.9 years) between July and October 2024 using an electronic survey. Included scales were the Diet Screening Tool, the US Adult Food Security Module, the International Physical Activity Questionnaire, the Social Connectedness Scale and the Depression, Anxiety and Stress Scale—21. Pearson’s correlation analyses and linear regression were performed exploring the associations for variables of interest.

**Results:**

The university student cohort was characterized by mild–severe depression (70%) and severe food insecurity (25%). The results showed increased food insecurity and physical activity (Metabolic Equivalent Task minutes/week) account for 11% and 9% of the variation seen in DASS-21 depression scores, respectively. Diet quality and social connections were not found to have a significant association with depressive symptomology.

**Conclusion:**

It appears that food insecurity could be a contributing factor impacting depressive symptomology, and if addressed effectively could improve mood disorder treatments. The relationship between physical activity and depression is counterintuitive and warrants further investigation. Implementing evidence-based holistic interventions that address food insecurity should be considered to support the mental health of university students.

## 1 Introduction

Young adults are at increased risk of depression and anxiety. Approximately 22% of 15–34-year-olds experienced these disorders in 2021 (1) and they are predicted to become the second largest cause of disability-adjusted life years (DALYs) by 2030 (2). Both biological and psychosocial factors are believed to be involved (3–6). Dietary interventions have the potential to influence biological pathways (7) affecting inflammation, oxidative stress, mitochondrial dysfunction, gut microbiota, tryptophan-kynurenine metabolism, hypothalamic–pituitary–adrenal (HPA) axis dysfunction, epigenetic changes, and neurogenesis (3, 7). Several of these biological pathways may also be influenced by food insecurity and physical activity. For instance, chronic stress associated with food insecurity can dysregulate the HPA axis (3), while physical activity has been shown to modulate this system and mitigate stress-related effects (4). Meta-analyses of epidemiological studies indicate that diet quality is inversely related to depression (8, 9). Factors with potential to influence diet quality for young adults, include food insecurity, physical activity, social connections and time availability (10, 11). Addressing and quantifying the contribution of factors that affect the interaction between diet quality and depression symptomology may offer avenues to better address the needs of this population. Food insecurity is defined as the inability to source adequate and safe food that meets nutritional needs in socially acceptable ways (5). Approximately 53% of Australian university students were classified as experiencing very low to marginal food security in 2025 up from 42% in 2022 (12). Food insecurity can prevent young adults from improving their diet quality (13). Experiencing food insecurity is also linked to increased risk ~40% of developing depressive symptomology in the general population (5). There may be an interaction between food insecurity and diet quality that explains the role they both have in development of depressive symptomology.

Physical activity may interact with diet quality with sedentary activity patterns associated with reduced consumption of healthy foods such as fruit and vegetables, impacting intake of nutrients such as fibre, calcium and vitamins (11). Participating in physical activity was also associated with decreased levels of depressive symptomology in young adults in a meta-analysis (4). However, studies examining the impact of various spheres of exercise, such as leisure, commuting, occupational and domestic duties, show varying effects on depression severity depending on the nature of the physical activity undertaken (14). Currently 5% of Australians meet the Australian Physical Activity and Sedentary Behavior Guidelines (2014) (15). Improved understanding of the interactions between diet quality, depression and physical activity has potential to improve options for depressive symptomology.

Social connections are defined as the extent of personal attachment and cooperation among social networks. Strong social connections have been linked to diet quality (16) and are considered to have implications for depression (6, 17). Dietary intake is influenced by the social norms of peer groups, with individual intake aligning with others within the social group (16). Social isolation can exacerbate depressive symptomology and result in further withdrawal leading to a vicious cycle, whereas good social support networks are associated with improved mental health outcomes (6).

University students are vulnerable to poor diet quality, food insecurity, inactivity, low social connections and depression. However, there is little understanding of the interaction between these factors within the university student population. The aim of this study was to analyze the relationships between diet quality, food insecurity, physical activity, social connectedness and depression in a cohort of Australian university students. The outcomes will identify areas of focus for future lifestyle and clinical support initiatives for students, and inform the design of collaborative research projects. We hypothesized that poorer diet quality, higher food insecurity, lower physical activity and lower social connectedness would be associated with greater depressive symptomology.

## 2 Method

### 2.1 Design

A cross-sectional, anonymous, electronic survey was conducted between July and October 2024, according to the Strengthening the Reporting of Observational Studies in Epidemiology (STROBE) Statement: guidelines for reporting observational studies (18). Ethics approval was granted by the University of Canberra Human Research Ethics Committee (HREC number 13692).

### 2.2 Procedure and participants

Australian adult (18 years+) University of Canberra students were invited to participate in this study via Facebook. Additional participants were sought through flyers on campus and through Sona systems, a teaching platform for undergraduate psychology students. Of the total sample, 8% were enrolled in psychology courses, with the remainder drawn from the broader student population. Participants were excluded if they were not currently undertaking study at an Australian university or were younger than 18 years. A minimum sample size of 68 was determined using G*Power (version 3.1.9.7), with effect size = 0.15, *α* = 0.05, and power = 0.8. Participants completed a survey hosted online through Qualtrics™, which included demographic details, measures of depressive symptomology, diet quality, social connections, food insecurity, and physical activity levels. The survey was piloted with six young adults prior to distribution to confirm functionality with no amendments required. Informed consent was given at commencement of the survey after the participants read the information sheet.

### 2.3 Assessments

Demographic characteristics including gender, year of birth, education, marital status and household income were collected. Height, body mass, smoking status, alcohol consumption, chronic disease, allergies, and mental health disorders were also self-reported.

The Depression, Anxiety and Stress Scale—21 Item (DASS-21) is 21-item scale measuring symptoms of depression, anxiety and stress with 7 items addressing each factor. The scale was not designed with an intention to assign patients to Diagnostic and Statistical Manual of Mental Disorders—V classifications, however cutoffs have been developed to represent worsening levels of mental health (19). Respondents were asked to consider their experiences over the previous 7 days and respond to the items on a 4-point Likert scale: (0) Did not apply to me at all; (1) Applied to me to some degree, or some of the time; (3) Applied to me to a considerable degree, or a good part of the time; and (4) Applied to me very much, or most of the time. An example question from the depression scale is “I could not seem to experience any positive feelings at all.” Scores range from 0 to 42 for each subscale, with scores from the depression subscale of 10–13 indicative of mild depression, 14–20 moderate depression, 21–27 severe depression, and 28 and above deemed extremely severe depression (20). The DASS-21 has demonstrated high validity and reliability (Depression *α* = 0.94, Anxiety *α* = 0.87, Stress *α* = 0.91), and correlates well with other established scales such as the Beck Depression Inventory (Depression *r* = 0.79, Anxiety *r* = 0.62, Stress *r* = 0.69) (20).

The Dietary Screening Tool (DST) evaluates diet quality and nutritional risk by probing intake of commonly consumed foods over a one-week period (21). The 25-item scale was originally designed to align with the 2005 Dietary Guidelines for Americans. To ensure relevancy for an Australian population some modifications were made, for example exchanging the words ‘lollies’ for ‘candy’ and ‘biscuits’ for ‘cookies’. Scoring was undertaken as outlined by Bailey et al. (21) with possible scores between 0 (low quality) to 105 (high quality). The DST was identified as suitable to assess diet quality in Australian populations (22). The test–retest reliability coefficient for the DST is 0.83 (*p* < 0.001) and has high validity (21).

The U.S. Adult Food Security Survey Module is designed to evaluate food insecurity and the prevalence of hunger in households (12). This validated screening tool measures the impact that lack of income may have on the amount and quality of food available, including anxiety over supply of food and skipping meals and has been used previously in Australian populations without amendment (12). Possible scores range from zero to ten and are categorized as follows: 0–2 Food secure; 3–4 Food insecure without hunger; 5–6 Food insecure with hunger (moderate); and 7 or more Food insecure with hunger (severe). The U.S. Adult Food Security Survey Module has demonstrated high sensitivity (98%) and specificity (92%) with good reliability (*α* = 0.87) (12).

Activity levels were measured by the International Physical Activity Questionnaire (IPAQ)—short form, a validated and widely used tool (23). The IPAQ assesses physical activity over the previous 7 days, detailing the level of intensity and duration of activity completed. Metabolic Equivalent of Task (MET) minutes per week, a weighted score that accounts for intensity and duration of physical activity, was calculated using the Guidelines for Data Processing and Analysis of the International Physical Activity Questionnaire (24). The IPAQ has a high test–retest reliability coefficient of 0.80 (*p* < 0.001) and a high reliability (23).

The Social Connectedness Scale—Revised (SCS-R) is a 20-item measure establishing participants self-perceived connections to others in social situations (17). Each item is measured on a 6-point Likert scale ranging from strongly disagree to strongly agree. An example statement includes “I feel close to people,” with negatively worded statements reverse-coded. Summed scores range from 20–120, with higher scores reflecting greater social connection. The SCS has high reliability (internal consistency *α* > 0.91), and validity (17).

### 2.4 Data analysis

Data analysis was conducted utilizing IBM Statistical Package for Social Sciences (SPSS, version 29.0.2.0). Missing values were calculated as per protocols for relevant scales where available, otherwise the mean estimated value was inputted. The continuous variables (age, BMI and IPAQ time sitting) were represented as mean ± standard deviation (SD). The remaining continuous variables (DST, DASS-21, SCS, and IPAQ MET minutes) were categorized and with the categorical variables (gender, education, marital status, household income, cigarette and alcohol use, chronic disease, allergies, and mental health status) represented as totals and percentages. Pearson’s correlation analyses were used to explore the association and to indicate potential significance of any relationship between depressive symptomology (dependent variable), and age, BMI, diet quality, social connections, food insecurity and physical activity. Linear regression analysis was conducted for all variables of interest (diet quality, physical activity, social connections and food insecurity) that were associated with DASS-21 (depression) scores. Potential confounders such as age and BMI were considered; they were not included in the final regression models due to a lack of meaningful associations with either the primary independent variable or the dependent variable (25). Including these variables in the models would have increased the risk of overfitting, particularly given the modest sample size and may have reduced statistical power without improving model validity (26). Variables were entered in a stepwise manner into the regression model. Standardized *β* coefficients were reported to facilitate comparison of predictor strength, consistent with common practice in psychological and health sciences where confidence intervals are not always reported when the focus is on relative influence rather than precise estimation (27).

## 3 Results

**Figure 1:**
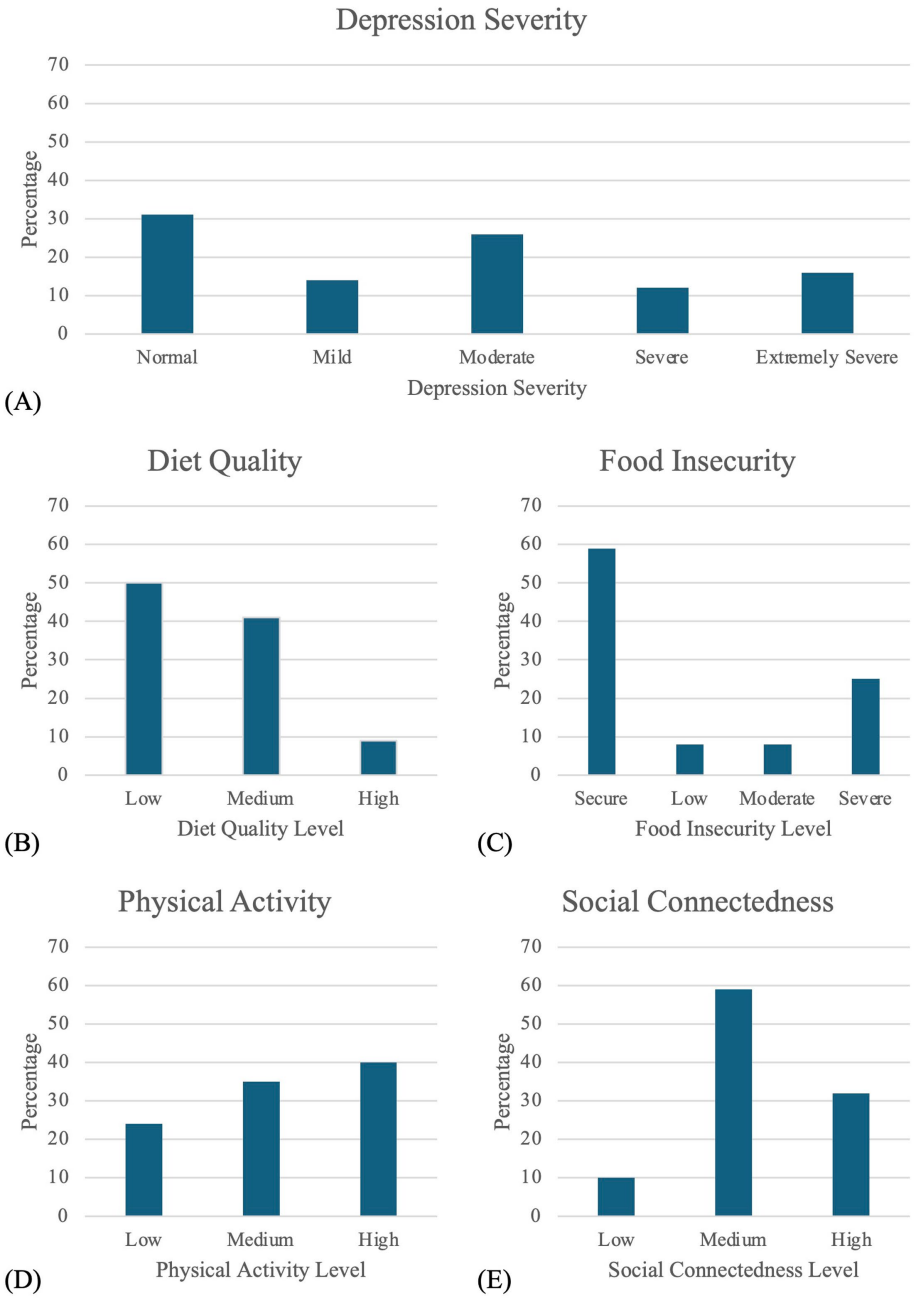
Distribution of participants across variables of interest. Graphs display the percentage of participants by category for **(A)** depression severity based on DASS scores, **(B)** diet quality, **(C)** food insecurity status, **(D)** physical activity level, and **(E)** social connectedness.

A total of 159 participants completed the initial screening for the survey, with 14 individuals excluded as they were not currently enrolled as university students, resulting in a total of 145 completed responses. Table 1; Figure 1 summarizes demographic and health measures for the participants.

**Table 1:** Participant details.

Demographic factors		Health factors	
*Gender (n, %)*		*Chronic disease (n, %)*	
Female	116 (80)	Yes	26 (18)
Male	22 (15)	No	113 (78)
Other	6 (4)	Non-disclosed	6 (4)
Non-disclosed	1 (1)		
		*Allergies and intolerances (n, %)*	
*Age (years; M, SD)*	26.9 (9.9)	Yes	36 (25)
		No	108 (74)
*BMI*^a^ *(kg/m^2^; M, SD)*	24.2 (6.6)	Non-disclosed	1 (1)
			
*Marital status (n, %)*		*Self-reported mental health (n, %)*	
Partnered	32 (22)	Depression	41 (28)
Not partnered	81 (80)	Other mental health diagnosis	42 (29)
Non-disclosed	5 (3)	No diagnosis	58 (40)
		Non-disclosed	4 (3)
*Household income per week (n, %)*			
Less than $1800	91 (63)	*DASS-21* ^b^ *—depression (n, %)*	
More than $1800	41 (28)	Normal	45 (31)
Non-disclosed	13 (9)	Mild	21 (14)
		Moderate	38 (26)
*Highest education level (n, %)*		Severe	18 (12)
Secondary school certificate	74 (51)	Extremely severe	23 (16)
Further education	67 (46)		
Non-disclosed	4 (3)	*DASS-21* ^b^ *—anxiety (n, %)*	
		Normal	35 (24)
*Smoking status (n, %)*		Mild	9 (6)
Yes	11 (8)	Moderate	35 (24)
No	134 (92)	Severe	18 (12)
		Extremely severe	48 (33)
*Alcohol (standard drinks/week; n, %)*			
None	73 (50)	*DASS-21* ^b^ *—stress (n, %)*	
1–3	38 (26)	Normal	52 (36)
4–6	11 (8)	Mild	23 (16)
7–9	17 (12)	Moderate	34 (23)
10 or more	5 (3)	Severe	25 (17)
Non-disclosed	1 (1)	Extremely severe	11 (8)
			
*Food insecurity score (n, %)*		*Diet quality (DST* ^c^ *) (n, %)*	
Food secure	86 (59)	Low	73 (50)
Low food insecurity	12 (8)	Medium	59 (41)
Moderate food insecurity	11 (8)	High	13 (9)
Severe food insecurity	36 (25)		
		*Social connection (SCS* ^d^ *) (n, %)*	
		Low	14 (10)
		Medium	85 (59)
		High	46 (32)
			
		*Physical activity (IPAQ* ^e^ *) (n, %)*	
		Low	24 (24)
		Medium	35 (35)
		High	40 (40)
		*Sedentary behavior (hours/day; M, SD)*	5.7 (1.6)

a BMI, body mass index.

b DASS-21, depression, anxiety and stress scale—21 items.

c DST, diet screening tool.

d SCS, social connectedness scale.

e IPAQ, international physical activity questionnaire.

The DASS-21 depression scale showed significant positive correlations with food insecurity, physical activity, anxiety and stress. Table 2 presents the Pearson’s correlation coefficients for all variables collected.

**Table 2:** Associations between depression, age, BMI, diet quality, social connections and food security reported as Pearson’s correlation [95% confidence interval].

	DASS-21^a^ (depression)	Age	BMI^b^	Dietary screening tool	Social connectedness scale	US adult food security module	International physical activity questionnaire	DASS-21^a^ (anxiety)
Age	−0.105 [−0.263, 0.059]							
BMI^b^	−0.053 [−0.214, 0.111]	0.299^**^ [0.142, 0.440]						
Dietary screening tool	−0.115 [−0.273, 0.049]	0.067 [−0.097, 0.228]	0.045 [−0.119, 0.207]					
Social connectedness scale	0.003 [−0.160, 0.166]	−0.075 [−0.235, 0.089]	−0.029 [−0.191, 0.135]	0.257^**^ [0.098, 0.403]				
US adult food security module	0.329^**^ [0.175, 0.467]	−0.081 [−0.241, 0.083]	−0.223^**^ [−0.372, −0.062]	0.042 [−0.122, 0.203]	0.042 [−0.122, 0.203]			
International physical activity questionnaire	0.304^**^ [0.098, 0.485]	−0.376^**^ [−0.544, −0.178]	−0.393^**^ [−0.5599, −0.198]	0.020 [−0.193, 0.231]	−0.078 [−0.285, 0.136]	0.651^**^ [0.509, 0.758]		
DASS-21^a^ (anxiety)	0.653^**^ [0.548, 0.737]	−0.224^**^ [−0.373, −0.063]	−0.085 [−0.245, 0.079]	−0.085 [−0.244, 0.079]	0.066 [−0.098, 0.226]	0.398^**^ [0.251, 0.527]	0.438^**^ [0.249, 0.594]	
DASS-21^a^ (stress)	0.662^**^ [0.559, 0.744]	−0.118 [−0.276, 0.046]	−0.033 [−0.195, 0.131]	−0.056 [−0.217, 0.108]	0.029 [−0.134, 0.191]	0.190^*^ [0.028, 0.342]	0.120 [−0.94, 0.323]	0.714^**^ [0.623, 0.785]

^*^p < 0.05, ^**^p < 0.01.

a DASS-21, depression, anxiety and stress scale—21 item scale.

b BMI, body mass index.

A linear regression analysis was performed for the two independent variables of interest, food insecurity and physical activity, that also correlated with depression scores on the DASS-21. Before interpreting the results, the assumptions of linearity, homoscedasticity, and multicollinearity were verified and met.

Food insecurity (Table 3) (*β* = 0.329, *t* = 4.159, *p* < 0.001, and physical activity, reported as METS minutes per week (Table 3) (*β* = 0.304, *t* = 2.923, *p* = 0.004) were positively associated with the degree of depressive symptomology. The explanatory variance was ~11% and ~9% for each variable, respectively. No other interactions were examined as they did not correlate significantly with DASS-21 (depression) scores.

**Table 3:** Regression analysis for factors of interest that correlated with depressive symptomology.

Factor		Standardised *β* coefficient	*F*	*p*-value	*R*	*R* ^2^	Adj *R*^2^
1	Food insecurity	0.329	17.300	<0.001	0.329	0.108	0.102
2	Physical activity	0.304	8.547	<0.004	0.304	0.092	0.082

## 4 Discussion

In our cohort of Australian university students (mean age 26.9 years) ~ 70% were categorized as having mild depression symptoms or greater compared with ~10% of Australians aged 25–34 years who experienced an affective disorder in 2020–2022 (1). The prevalence of food insecurity in this cohort was ~41% comprising ~8% low food insecurity, ~8%, moderate food insecurity and ~25% severe food insecurity which overall aligns with other findings addressing the prevalence of food insecurity in Australian university student populations in 2025 (12). However the levels of severe food insecurity have increased from 17% in 2022 to 29% in 2025. The results of the current study align with these findings with 25% of this cohort experiencing severe food insecurity. The Australian Physical Activity and Sedentary Behavior Guidelines recommend 150–300 min of moderate intensity activity or 75–150 min of high intensity activity per week which equates to 600–1200 MET minutes per week. This requirement was met by 88% of the participants in this study. Increased levels of food insecurity and higher MET minutes per week were associated with increased depression scores on the DASS-21, explaining ~11% and ~9% of the variation in depressive symptomology, respectively. Diet quality did not explain any of the variation in depressive symptoms contrasting with our expectations and the findings from systematic reviews of the current literature (8, 9).

The lack of association between diet quality and depression in a university student cohort has recently been identified by several other studies. Myrissa et al. (28) observed no correlation between diet quality and depression in UK undergraduate students. Examination of five different dietary patterns in Lebanese university students reported no significant associations with depression after controlling for confounders (29). Investigations of the link(s) between inflammatory dietary intake and mental health in female collegiate athletes also noted increased diet quality was associated with worse mental health (30). No relationship was evident between diet quality and depression in male university students in three European countries (31). There are several possible explanations for this unexpected finding. There is a large heterogeneity in how diet is measured and depression is assessed in the current studies (32). Variation exists in the choice of tools for measuring diet quality with studies using a mix of food frequency questionnaires and diet quality measures. Diet quality measures allow for a more straightforward interpretation of how closely dietary intake aligns with guidelines, resulting in easier analysis (22). Similarly a wide variety of validated scales are used to measure depressive symptomology (28). Inconsistencies in diet quality measures and depression scales may have influenced the assessment of the relationship between diet quality and depressive symptomology (32). Finally, some studies identified that different demographics, such as gender and age, have different outcomes for the relationship between diet quality and depressive symptomology (30, 31) creating further complexities that need to be addressed when assessing the impact of diet quality on depressive symptomology.

Food insecurity impacts mental health by increasing stress and negatively impacting diet quality. The primary causes of food insecurity include reduced income, challenges accessing healthy food, and lack of education about healthy food and its preparation (33). A systematic review and meta-analysis concluded that food insecurity has a direct impact on depression (OR = 1.40, 95%CI: 1.30–1.58) and stress (OR = 1.34, 95%CI: 1.24–1.44) (5). University students living with food insecurity often face increased levels of stress due to limited access to healthy food, fear of running out of food, or making significant changes to their dietary preferences (34). Food insecurity has been associated with increased intake of fats, refined sugars and salt, and reduced intakes of fruits, vegetables and fibre are associated with food insecurity (35). Such a dietary pattern is associated with a poor quality diet (36) and has been linked to increased depressive symptomology (37) potentially explaining in part the relationship between food insecurity and depression. This dietary pattern is also associated with nutrient deficiencies which may compound the impact of poor diet quality on depressive symptomology (38). While diet quality did not directly correlate with depressive symptoms in this study, it remains a consideration given the limitations outlined earlier. A cross-sectional study on Australian university students reported that food insecurity negatively affects diet quality (39). Students with excellent cooking skills who cooked for themselves more frequently were more food secure (39). This finding aligns with studies with American college students detailing that as food insecurity increased cooking self-efficacy and food preparation scores decrease (13). Particularly for individuals with low income, food insecurity resulting from a lack of education about healthy food and its preparation may further exacerbate mental health issues by promoting poor dietary practices (40). Food insecurity also may impact on social connections causing feelings of isolation (34, 38) resulting in increased risk of depression (41).

Our physical activity findings contradict the well-accepted understanding that physical activity has a protective effect on depressive symptomology (42). In this cohort higher MET minutes per week was associated with greater severity in depressive symptomology. Ansari et al. (43) also found a similar effect. This deviation from the typical findings may be explained by the nature (type and setting) of the physical activity undertaken (14, 44, 45). Physical activity that is undertaken as part of an individual’s occupation has been associated with increased severity of depressive symptomology (14, 44). Furthermore lower socioeconomic status in women undertaking occupationally-linked physical activity has been associated with poorer mental health (45) which becomes an important consideration with 80% of the participants in this study identifying as female and 91% with household incomes of less than $AUD 1800 per week. Physical activity undertaken as household chores also may impact depending upon the gender of the individual with this type of physical activity associated with a reduced risk of depressive symptoms for men (44). Further research is required to examine the relationship between physical activity and depressive symptoms. It is recommended that analyses be conducted by the type of physical activity, such as occupational, domestic, transport-related, or leisure-time activity, as these domains may differ in their psychological and physiological implications.

Shifts in the composition of the gut microbiota have also been linked to increased depressive symptomology (7). Physical activity can impact the gut microbiota, with extended periods of high intensity exercise associated with disruptions to the gut microbiota and increased inflammation (46). The increased amount of physical activity undertaken by this cohort may be causing gut microbiota dysbiosis and higher levels of inflammation which has also been associated with worsening depressive symptomology (47). Gut microbiota dysbiosis might explain the link between physical activity and depressive symptoms identified in this cohort. Finally, links have been made between low energy availability and increased risk of mood disorders (48). Athletes with low energy availability were noted to be 2.3 times more likely to report feelings of depression compared to athletes with adequate energy availability (49). This cohort may be experiencing low energy availability due to the reported high MET minutes and low-quality diet suggesting another potential explanation for our findings. Further research is needed to better understand the interactions between physical activity and depressive symptomology.

Lifestyle changes are increasingly promoted to improve treatment outcomes for individuals living with MDD. Current guidelines for treatment of mood disorders recommend lifestyle modification, including diet, as a first line intervention (50). The current findings highlight the need for targeted campus-based interventions. Strategies such as university food pantries, subsidized meal programs, and cooking workshops could help alleviate food insecurity and promote healthier eating habits and improved mental health for students. To ensure effective implementation of these initiatives, collaboration with relevant dietitians and other allied health professionals is essential (51). Dietary interventions facilitated by qualified professionals are associated with improved outcomes given their unique skill set for addressing the multifaceted issues that arise through increased food insecurity, general poor diet quality and special nutritional needs (52). Ensuring all individuals with poor mental health have access to dietitian-directed programs to guide their dietary changes is a policy aspect needing attention (53).

### 4.1 Limitations

Limitations of this study include the reliance on self-reported data. As no clinical assessment was undertaken it is difficult to determine the severity of depressive symptomology associated with diet quality, and how individuals perceive their own experiences (54). There is also a lack of generalizability of the results. Participant recruitment through social media and psychology teaching platforms may have introduced selection bias, potentially limiting the generalization of findings to the university student population. Additionally, the focus on a student population further limits generalizability to cohorts and populations other than young adults studying at university. Their unique circumstances, for example living out of home for the first time, stresses of study and balancing other life commitments, may not be directly relevant to other populations. Finally, given the cross-sectional nature of this study causality cannot be determined. Additionally, while the regression model examined dietary and social variables of primary interest, residual confounding factors not addressed here may have had some underlying influence. Factors, such as income variability, were not fully captured and may have contributed to the observed associations. Additionally, the sample size is relatively small, which may limit the reliability of the results, particularly for the regression and correlation analyses and increases the potential for random variation to influence the observed associations (55, 56). Future research should evaluate dietary interventions that address food insecurity. Priorities include interventions that incorporate group cooking classes to address lack of education around healthy food preparation while promoting improved social connections. Special consideration to the types of recipes prepared with a focus on vegetarian options or use of legumes as a low-cost protein alternative may also help address both the economic considerations and further improve food security.

## 5 Conclusion

There are connections between food insecurity, increased duration and intensity of physical activity and depressive symptomology in a cohort of young adult university students. Diet quality and social connectedness were not significantly associated with depressive symptomology in this sample, though they both remain important targets for future research. Developing evidence-based interventions that are sympathetic to these contributing factors will enhance clinicians’ ability to support individuals living with MDD and improve treatment outcomes. All professionals working within the mental health space need to advocate for a holistic approach to support those individuals. Improvements to mental health through food access and security will open alternative avenues for treatment for those who need them.

## Data Availability

The datasets presented in this article are not available due to restrictions by ethics committee to address privacy and ethical considerations. Requests to access the datasets should be directed to the corresponding author.

## References

[1] Australian Bureau of Statistics. National study of mental health and wellbeing. Australian government (2023). Available online at: https://www.abs.gov.au/statistics/health/mental-health/national-study-mental-health-and-wellbeing/latest-release (accessed August 27, 2024)

[2] Mathers CD Loncar D. Projections of global mortality and burden of disease from 2002 to 2030. PLoS Med. (2006) 3:e442. doi: 10.1371/journal.pmed.0030442, PMID: 17132052 PMC1664601

[3] Malhi GS Mann JJ. Depression. Lancet. (2018) 392:2299. doi: 10.1016/S0140-6736(18)31948-2 30396512

[4] Bailey AP Hetrick SE Rosenbaum S Purcell R Parker AG. Treating depression with physical activity in adolescents and young adults: a systematic review and meta-analysis of randomised controlled trials. Psychol Med. (2018) 48:1068–83. doi: 10.1017/S0033291717002653, PMID: 28994355

[5] Pourmotabbed A Moradi S Babaei A Ghavami A Mohammadi H Jalili C. Food insecurity and mental health: a systematic review and meta-analysis. Public Health Nutr. (2020) 23:1778–90. doi: 10.1017/S136898001900435X, PMID: 32174292 PMC10200655

[6] Thoits PA. Mechanisms linking social ties and support to physical and mental health. J Health Soc Behav. (2011) 52:145–61. doi: 10.1177/0022146510395592, PMID: 21673143

[7] Marx W Lane M Hockey M Aslam H Berk M Walder K. Diet and depression: exploring the biological mechanisms of action. Mol Psychiatry. (2021) 26:134–50. doi: 10.1038/s41380-020-00925-x, PMID: 33144709

[8] Lassale C Batty GD Baghdadli A Jacka F Sánchez-Villegas A Kivimäki M. Healthy dietary indices and risk of depressive outcomes: a systematic review and meta-analysis of observational studies. Mol Psychiatry. (2019) 24:965–86. doi: 10.1038/s41380-018-0237-8, PMID: 30254236 PMC6755986

[9] Wu P-Y Lin M-Y Tsai P-S. Alternate healthy eating index and risk of depression: a meta-analysis and systemematic review. Nutr Neurosci. (2020) 23:101–9. doi: 10.1080/1028415X.2018.1477424, PMID: 29804517

[10] Sexton-Dhamu MJ Livingstone KM Pendergast FJ Worsley A McNaughton SA. Individual, social–environmental and physical–environmental correlates of diet quality in young adults aged 18–30 years. Appetite. (2021) 162:105175. doi: 10.1016/j.appet.2021.105175, PMID: 33640428

[11] Gillman MW Pinto BM Tennstedt S Glanz K Marcus B Friedman RH. Relationships of physical activity with dietary behaviors among adults. Prev Med. (2001) 32:295–301. doi: 10.1006/pmed.2000.0812, PMID: 11277687

[12] Kent K Visentin D Peterson C Elliott C Primo C Murray S. Food insecurity among Australian university students is higher and more severe across an extended period of high inflation: a repeated cross-sectional study 2022-2024. Health Promot J Austr. (2025) 36:e70037. doi: 10.1002/hpja.70037, PMID: 40195071 PMC11976040

[13] Knol LL Robb CA McKinley EM Wood M. Very low food security status is related to lower cooking self-efficacy and less frequent food preparation behaviors among college students. J Nutr Educ Behav. (2019) 51:357–63. doi: 10.1016/j.jneb.2018.10.009, PMID: 30528982

[14] Werneck AO Stubbs B Szwarcwald CL Silva DR. Independent relationships between different domains of physical activity and depressive symptoms among 60,202 Brazilian adults. Gen Hosp Psychiatry. (2020) 64:26–32. doi: 10.1016/j.genhosppsych.2020.01.007, PMID: 32086172

[15] Australian Bureau of Statistics (ABS). Physical activity (2022). Available online at: https://www.abs.gov.au/statistics/health/health-conditions-and-risks/physical-activity/latest-release#:~:text=One%20in%20twenty%20(5.6%25),2017%E2%80%9318%20(1.9%25) (accessed October 14, 2024)

[16] Neely E Walton M Stephens C. Young people's food practices and social relationships. A thematic synthesis. Appetite. (2014) 82:50–60. doi: 10.1016/j.appet.2014.07.005, PMID: 25017130

[17] Lee RM Robbins SB. Measuring belongingness: the social connectedness and the social assurance scales. J Couns Psychol. (1995) 42:232–41. doi: 10.1037/0022-0167.42.2.232

[18] Von Elm E Altman DG Egger M Pocock SJ Gøtzsche PC Vandenbroucke JP. The strengthening the reporting of observational studies in epidemiology (STROBE) statement: guidelines for reporting observational studies. Int J Surg. (2014) 12:1495–9. doi: 10.1016/j.ijsu.2014.07.013, PMID: 25046131

[19] McDowell I. Measuring health: a guide to rating scales and questionnaires. 3rd ed. New York: Oxford University Press (2006).

[20] Antony MM Bieling PJ Cox BJ Enns MW Swinson RP. Psychometric properties of the 42-item and 21-item versions of the depression anxiety stress scales in clinical groups and a community sample. Psychol Assess. (1998) 10:176–81. doi: 10.1037/1040-3590.10.2.176

[21] Bailey RL Miller PE Mitchell DC Hartman TJ Lawrence FR Sempos CT. Dietary screening tool identifies nutritional risk in older adults. Am J Clin Nutr. (2009) 90:177–83. doi: 10.3945/ajcn.2008.27268, PMID: 19458013 PMC2697000

[22] Tan MS Cheung HC McAuley E Ross LJ MacLaughlin HL. Quality and validity of diet quality indices for use in Australian contexts: a systematic review. Br J Nutr. (2022) 128:2021–45. doi: 10.1017/S0007114521004943, PMID: 34913425

[23] Craig CL Marshall AL Sjöström M Bauman AE Booth ML Ainsworth BE. International physical activity questionnaire: 12-country reliability and validity. Med Sci Sports Exerc. (2003) 35:1381–95. doi: 10.1249/01.MSS.0000078924.61453.FB, PMID: 12900694

[24] Forde C. Guidelines for data processing and analysis of the International Physical Activity Quesionnaire (IPAQ)—short and long forms. Available online at: https://ugc.futurelearn.com/uploads/files/bc/c5/bcc53b14-ec1e-4d90-88e3-1568682f32ae/IPAQ_PDF.pdf (accessed January 15, 2025).

[25] VanderWeele TJ. Principles of confounder selection. Eur J Epidemiol. (2019) 34:211–9. doi: 10.1007/s10654-019-00494-6, PMID: 30840181 PMC6447501

[26] Mazor Y. What you see is not what you get: when unity masquerades as disarray. Scand J Old Testam. (2006) 20:264–72. doi: 10.1080/09018320601049532

[27] Fernández-Castilla B Aloe AM Declercq L Jamshidi L Onghena P Natasha Beretvas S. Concealed correlations meta-analysis: a new method for synthesizing standardized regression coefficients. Behav Res Methods. (2019) 51:316–31. doi: 10.3758/s13428-018-1123-7, PMID: 30251007

[28] Myrissa K Court C Kelaiditi E. Cross-sectional study examining the association between diet quality and the prevalence of anxiety and depression in UK undergraduate students. Nutr Bull. (2024) 49:383–95. doi: 10.1111/nbu.12694, PMID: 38940391

[29] Jaalouk D Matar Boumosleh J Helou L Abou Jaoude M. Dietary patterns, their covariates, and associations with severity of depressive symptoms among university students in Lebanon: a cross-sectional study. Eur J Nutr. (2019) 58:997–1008. doi: 10.1007/s00394-018-1614-4, PMID: 29352383

[30] Christensen N van Woerden I Aubuchon-Endsley NL Fleckenstein P Olsen J Blanton C. Diet quality and mental health status among division 1 female collegiate athletes during the COVID-19 pandemic. Int J Environ Res Public Health. (2021) 18:13377. doi: 10.3390/ijerph182413377, PMID: 34948985 PMC8703292

[31] Mikolajczyk RT El Ansari W Maxwell AE. Food consumption frequency and perceived stress and depressive symptoms among students in three European countries. Nutr J. (2009) 8:31. doi: 10.1186/1475-2891-8-31, PMID: 19604384 PMC2716364

[32] Quirk SE Williams LJ O'Neil A Pasco JA Jacka FN Housden S. The association between diet quality, dietary patterns and depression in adults: a systematic review. BMC Psychiatry. (2013) 13:175. doi: 10.1186/1471-244X-13-175 23802679 PMC3706241

[33] Seivwright AN Callis Z Flatau P. Food insecurity and socioeconomic disadvantage in Australia. Int J Environ ResPublic Health. (2020) 17:559. doi: 10.3390/ijerph17020559, PMID: 31952327 PMC7014009

[34] Meza A Altman E Martinez S Leung CW. “It’s a feeling that one is not worth food”: a qualitative study exploring the psychosocial experience and academic consequences of food insecurity among college students. J Acad Nutr Diet. (2019) 119:1713–1721.e1. doi: 10.1016/j.jand.2018.09.006, PMID: 30553586 PMC6561835

[35] Melchior M Chastang J-F Falissard B Galéra C Tremblay RE Côté SM. Food insecurity and children's mental health: a prospective birth cohort study. PLoS One. (2012) 7:e52615. doi: 10.1371/journal.pone.0052615, PMID: 23300723 PMC3530436

[36] McAuley EA MacLaughlin HL Hannan-Jones MT King N Ross LJ. Effectiveness of diet quality indices in measuring a change in diet quality over time: a systematic review and meta-analysis of randomized controlled trials. Nutr Rev. (2023) 81:361–83. doi: 10.1093/nutrit/nuac063, PMID: 36102824

[37] Jacka FN. Nutritional psychiatry: where to next? EBioMedicine. (2017) 17:24–9. doi: 10.1016/j.ebiom.2017.02.020, PMID: 28242200 PMC5360575

[38] Palar K Kushel M Frongillo EA Riley ED Grede N Bangsberg D. Food insecurity is longitudinally associated with depressive symptoms among homeless and marginally-housed individuals living with HIV. AIDS Behav. (2015) 19:1527–34. doi: 10.1007/s10461-014-0922-9, PMID: 25351185 PMC4414669

[39] Shi Y Grech A Allman-Farinelli M. Diet quality among students attending an Australian university is compromised by food insecurity and less frequent intake of home cooked meals. a cross-sectional survey using the validated healthy eating index for Australian adults (HEIFA-2013). Nutrients. (2022) 14:4522. doi: 10.3390/nu14214522, PMID: 36364787 PMC9655026

[40] Kohanmoo A Hashemzadeh M Teymouri M Zare M Akhlaghi M. Food insecurity is associated with low diet quality and unhealthy cooking and eating habits in Iranian women. J Health Popul Nutr. (2024) 43:42. doi: 10.1186/s41043-024-00533-3, PMID: 38486251 PMC10941397

[41] Matthews T Danese A Wertz J Odgers CL Ambler A Moffitt TE. Social isolation, loneliness and depression in young adulthood: a behavioural genetic analysis. Soc Psychiatry Psychiatr Epidemiol. (2016) 51:339–48. doi: 10.1007/s00127-016-1178-7, PMID: 26843197 PMC4819590

[42] Schuch FB Vancampfort D Firth J Rosenbaum S Ward PB Silva ES. Physical activity and incident depression: a meta-analysis of prospective cohort studies. Am J Psychiatry. (2018) 175:631–48. doi: 10.1176/appi.ajp.2018.17111194, PMID: 29690792

[43] Ansari A Karimi K Rashidi F Memari A Salehi S Danandeh K. Physical activity and its specific domains associated with depressive symptoms: a cross-sectional large population survey. Int J Surg Glob Health. (2025) 8:e00557. doi: 10.1097/GH9.0000000000000557

[44] Qie R Huang H Sun P Wu J Ba Y Zhou G. Physical activity domains and patterns with risk of depressive symptoms: a cross-sectional study in China. J Affect Disord. (2023) 337:120–7. doi: 10.1016/j.jad.2023.05.091, PMID: 37263360

[45] Teno SC Silva MN Júdice PB. Physical activity and sedentary behaviour-specific domains and their associations with mental health in adults: a systematic review. Adv Ment Health. (2024) 22:738–65. doi: 10.1080/18387357.2024.2324099

[46] Varghese S Rao S Khattak A Zamir F Chaari A. Physical exercise and the gut microbiome: a bidirectional relationship influencing health and performance. Nutrients. (2024) 16:3663. doi: 10.3390/nu16213663, PMID: 39519496 PMC11547208

[47] Carlessi AS Borba LA Zugno AI Quevedo J Reus GZ. Gut microbiota-brain axis in depression: the role of neuroinflammation. Eur J Neurosci. (2021) 53:222–35. doi: 10.1111/ejn.14631 31785168

[48] Mountjoy M Ackerman KE Bailey DM Burke LM Constantini N Hackney AC. 2023 International Olympic Committee’s (IOC) consensus statement on relative energy deficiency in sport (REDs). Br J Sports Med. (2023) 57:1073–98. doi: 10.1136/bjsports-2023-106994, PMID: 37752011

[49] Ackerman KE Holtzman B Cooper KM Flynn EF Bruinvels G Tenforde AS. Low energy availability surrogates correlate with health and performance consequences of relative energy deficiency in sport. Br J Sports Med. (2019) 53:628–33. doi: 10.1136/bjsports-2017-098958, PMID: 29860237

[50] Malhi GS Bell E Bassett D Boyce P Bryant R Hazell P. The 2020 Royal Australian and new Zealand College of Psychiatrists clinical practice guidelines for mood disorders. Aust N Z J Psychiatry. (2021) 55:7–117. doi: 10.1177/0004867420979353, PMID: 33353391

[51] Teasdale SB Latimer G Byron A Schuldt V Pizzinga J Plain J. Expanding collaborative care: integrating the role of dietitians and nutrition interventions in services for people with mental illness. Australas Psychiatry. (2018) 26:47–9. doi: 10.1177/1039856217726690, PMID: 28869391

[52] Holmes AL Sanderson B Maisiak R Brown A Bittner V. Dietitian services are associated with improved patient outcomes and the MEDFICTS dietary assessment questionnaire is a suitable outcome measure in cardiac rehabilitation. J Am Diet Assoc. (2005) 105:1533–40. doi: 10.1016/j.jada.2005.08.001, PMID: 16183352

[53] Dietitians Australia. Dietitians Australia mental health evidence brief 2024. Canberra, Australia: (2024). Available online at: https://dietitiansaustralia.org.au/sites/default/files/2024-04/Dietitians%20Australia%20Mental%20Health%20Evidence%20Brief%202024.pdf (accessed April 28, 2025).

[54] Hobbs C Lewis G Dowrick C Kounali D Peters TJ Lewis G. Comparison between self-administered depression questionnaires and patients' own views of changes in their mood: a prospective cohort study in primary care. Psychol Med. (2021) 51:853–60. doi: 10.1017/S0033291719003878, PMID: 31957623 PMC8108392

[55] Fraley RC Vazire S. The N-pact factor: evaluating the quality of empirical journals with respect to sample size and statistical power. PLoS One. (2014) 9:e109019. doi: 10.1371/journal.pone.0109019, PMID: 25296159 PMC4189949

[56] Schönbrodt FD Perugini M. At what sample size do correlations stabilize? J Res Pers. (2013) 47:609–12. doi: 10.1016/j.jrp.2013.05.009

